# Cognitive phenotype and neurodegeneration associated with Tau in Huntington's disease

**DOI:** 10.1002/acn3.52031

**Published:** 2024-03-27

**Authors:** Saul Martinez‐Horta, Jesús Perez‐Perez, Rocío Perez‐Gonzalez, Frederic Sampedro, Andrea Horta‐Barba, Antonia Campolongo, Elisa Rivas‐Asensio, Arnau Puig‐Davi, Javier Pagonabarraga, Jaime Kulisevsky

**Affiliations:** ^1^ Movement Disorders Unit, Neurology Department Hospital de la Santa Creu i Sant Pau Barcelona Spain; ^2^ Biomedical Research Institute Sant Pau (IIB‐Sant Pau) Barcelona Spain; ^3^ Centro de Investigación Biomédica en Red‐Enfermedades Neurodegenerativas (CIBERNED) Madrid Spain; ^4^ Department of Medicine Autonomous University of Barcelona Barcelona Spain; ^5^ European Huntington's Disease Network (EHDN); ^6^ Instituto de Investigación Sanitaria y Biomédica de Alicante (ISABIAL) and Instituto de Neurociencias UMH‐CSIC Alicante Spain; ^7^ Neuroradiology unit, Radiology Department Hospital de la Santa Creu i Sant Pau Barcelona Spain

## Abstract

**Objective:**

The clinical phenotype of Huntington's disease (HD) can be very heterogeneous between patients, even when they share equivalent CAG repeat length, age, or disease burden. This heterogeneity is especially evident in terms of the cognitive profile and related brain changes. To shed light on the mechanisms participating in this heterogeneity, the present study delves into the association between Tau pathology and more severe cognitive phenotypes and brain damage in HD.

**Methods:**

We used a comprehensive neuropsychological examination to characterize the cognitive phenotype of a sample of 30 participants with early‐to‐middle HD for which we also obtained 3 T structural magnetic resonance image (MRI) and cerebrospinal fluid (CSF). We quantified CSF levels of neurofilament light chain (NfL), total Tau (tTau), and phosphorylated Tau‐231 (pTau‐231). Thanks to the cognitive characterization carried out, we subsequently explored the relationship between different levels of biomarkers, the cognitive phenotype, and brain integrity.

**Results:**

The results confirmed that more severe forms of cognitive deterioration in HD extend beyond executive dysfunction and affect processes with clear posterior‐cortical dependence. This phenotype was in turn associated with higher CSF levels of tTau and pTau‐231 and to a more pronounced pattern of posterior‐cortical atrophy in specific brain regions closely linked to the cognitive processes affected by Tau.

**Interpretation:**

Our findings reinforce the association between Tau pathology, cognition, and neurodegeneration in HD, emphasizing the need to explore the role of Tau in the cognitive heterogeneity of the disease.

## Introduction

Huntington's disease (HD) associates a progressive cognitive decline that has historically been considered prototypical of a subcortical dementia due to the characteristic pattern of striatal atrophy of the disease.^1^, ^2^, ^3^, ^4^, ^5^ Accordingly, cognitive deficits mostly attributable to frontal‐executive, attention, and processing speed disturbances affect most HD patients from the very beginning of the disease and progressively worsen to the point of dementia.^6^, ^7^

Damage at the level of basal ganglia unquestionably play a cardinal role in the general cognitive phenotype of HD. However, it is indisputable that HD also associates impairment in other cognitive domains, and that the severity of these alterations is mediated by the dysfunction of systems not necessarily dependent on striatal integrity.^3^, ^8^, ^9^, ^10^, ^11^, ^12^, ^13^, ^14^, ^15^, ^16^, ^17^, ^18^

HD is genetically determined by a well‐known single mutation in the HTT gene.^4^ Despite the recognized relationship between the size of the CAG expansion and age of onset, clinical phenotype, and disease severity, there is a remarkably significant variability among patients who share the same CAG expansion size, age, or disease burden. Variability is also observed in terms of the pattern of brain damage and in the neurocognitive profile exhibited by patients.^1^, ^2^, ^19^ This suggests that, beyond the effect directly mediated by mutant HTT (mHTT), other neuropathological mechanisms could variably affect patients and contribute to the clinical and cognitive heterogeneity exhibited by HD individuals. In this sense, in recent years evidence has been accumulating on different mechanisms involved in this variability, highlighting the role of genetic modifiers, among which those that contribute to the failure of DNA repair mechanisms and somatic instability are particularly relevant.^20^

Beyond these mechanisms, Tau‐related pathology has been proposed as one of the possible additional mechanisms that could contribute, in a synergistical manner, to the cognitive heterogeneity of HD.^21^ Early studies on the potential role of Tau protein in HD showed Tau pathology in Braak stages I‐III in 60% of cases. Subsequently, it was demonstrated that 80% of patients with HD and associated dementia exhibited severe Tau pathology consistent with Braak stages V‐VI.^22^, ^23^ More recent studies have revealed elevated levels of Tau protein in the cerebrospinal fluid (CSF) of HD patients, similar to those reported in Alzheimer's disease.^24^ Although this might suggest that HD patients have Alzheimer's disease‐like copathology, additional works have shown that mHTT promotes neurodegeneration through a direct interaction with Tau protein which induces changes in protein structure, Tau phosphorylation, and 4R/3R Tau deposition.^25^, ^26^ All these findings have led to the proposal of incorporating HD into the group of secondary tauopathies.^27^

In the present study, we hypothesize that in HD, Tau pathology contributes to the expression of more severe cognitive phenotypes being in turn associated with a more severe and widespread pattern of brain damage. To test this hypothesis, we specifically looked at CSF levels of neurofilament light chain (NfL), total Tau (tTau), and pTau‐231 (pTau) in a cohort of symptomatic HD patients who performed a comprehensive neuropsychological assessment and 3 T MRI acquisition.

## Materials and Methods

### Participants

We assessed 30 symptomatic gene‐mutation carriers (CAG > 39) regularly attending the outpatient HD‐Clinic of the Movement Disorders Unit at Hospital de la Santa Creu i Sant Pau in Barcelona. All participants were free of any neurological disorder other than HD and had no history of brain surgery, traumatic brain injury, epilepsy, drug abuse, or uncompensated systemic disease. For comparison purposes at biomarkers levels, we also included a sample of 22 asymptomatic carriers.

Written informed consent was obtained from all participants, and all procedures were performed in accordance with the standards of the Ethics Committee at Hospital de la Santa Creu i Sant Pau in Barcelona and in accordance with the 1964 Declaration of Helsinki and its later amendments.

### Assessments

The Unified Huntington's Disease Rating Scale—Total motor score (UHDRS‐TMS) was administered to rate the severity of motor symptoms. All patients were assigned a diagnostic confidence level (DCL) of 4 and classified as early or mild disease stage according to the total functional capacity (TFC) score (TFC > 11 for Stage I and TFC between 6 and 11 for Stage II).^28^ For the subgroup of premanifest participants, the diagnostic category was based on an DCL <3 and a TFC = 13.

The CAG age product (CAP score), an index assumed to reflect lifelong exposure to mutant huntingtin, was calculated using the following formula based on age and CAG repeat length: CAP = age × (CAG‐33.5).^29^ General clinical and sociodemographic data were also recorded.

### Neuropsychological assessment

A comprehensive neuropsychological assessment was administered to all manifest participants. Global cognition was assessed using the Parkinson's Disease–Cognitive Rating Scale (PD‐CRS).^30^ This instrument has been proved to exhibit very good psychometric properties in the context of HD.^31^

The cognitive domains covered by the comprehensive neuropsychological assessment were memory, attention and working memory, language, processing speed, visuospatial and visuoconstructive functions, and executive functions. Each cognitive domain was assessed at least with two different tests. Raw scores were corrected for age and education and transformed to *z* scores based on normative data.

For the memory domain, episodic memory was assessed using the Free and Cued Selective Reminding Test (FCSRT).^32^ Visual memory was assessed with the delayed recall of the Rey Osterrieth Complex Figure (ROCF).^33^ Attention and working memory were assessed using the forward and backward digit‐span subtest from the WAIS‐III, and with the part A of the Trail Making Test (TMT).^34^ For the language domain, we used Boston Naming Test (BNT‐60) for naming and the semantic fluency test. Visuoconstructive, visuoperceptive, and visuospatial functions were assessed with the copy of the ROCF, the Judgment of Line Orientation Test (JLOT), the Benton's Facial Recognition test, and with the subtests of position discrimination and number location of the Visual Object and Shape Perception Test (VOSP).^35^ Executive functions and processing speed were covered with the Symbol Digit Modalities Test (SDMT), the phonetic verbal fluency test, the Stroop test, and with the part B of the TMT.^34^, ^36^

### Biosample collection and processing

The CSF samples were collected and processed according to international recommendations. The CSF total Tau was measured with the Neurology 3‐Plex Advantage (Cat# 101995) and pTau‐231 with Advantage kit (Cat# 102292) respectively, using single molecule array technology (Simoa; Quanterix, Lexington, MA, USA). Samples were incubated first with the specific antibody‐coated paramagnetic beads and after a wash with the biotinylated antibody detector. After a second wash, streptavidin‐conjugated β‐galactosidase (SBG) reagent was added to bind the biotinylated antibodies, leading to the SBG enzyme labeling of the capture of total Tau or pTau. Once in the SR‐X platform, the beads were resuspended in resorufin β‐D‐galactopyranoside (RGP) reagent and transferred to the Simoa disk and sealed. Total Tau and pTau‐231 were then captured by the antibody‐coated paramagnetic beads and labeled with the SBG reagent and hydrolyzed the RGP substrate to produce a fluorescent signal. The fluorescent signal was generated from the calibration curve of known concentrations and fit using a 4‐parameter logistic curve that will be used to calculate the unknown and control samples concentrations. From among the existing set of pTau epitopes, the choice of pTau‐231 was due to the fact that pTau‐231 showed a high sensitivity to detect changes prior to tangle pathology formation, as well as to discriminate Braak stages 0 to 1.^37^, ^38^, ^39^

### Neuroimaging acquisition and preprocessing

T1‐weighted MRI images were acquired on a 3 T Philips Achieva using an MP‐RAGE sequence (TR/TE = 12.66/7.11 milliseconds, flip‐angle = 8°, field of view = 23 cm, matrix = 256 × 256, and slice thickness = 1 mm). We applied standard voxel‐based morphometry (VBM) and cortical thickness (Cth) neuroimaging pipelines. VBM procedures were performed using the statistical parametrical mapping software package (SPM12, http://www.fil.ion.ucl.ac.uk/spm). Briefly, GMV tissue probability maps were computed from T1‐MRI scans. These maps were then normalized to the Montreal Neurological Institute (MNI) space by applying the DARTEL algorithm. The resulting normalized GMV maps were then smoothed using an isotropic spatial filter of 8 × 8 × 8 mm full‐width at half‐maximum (FWHM) to reduce inter‐individual variability. Cth analysis was performed using the FreeSurfer 6.0 software package (https://surfer.nmr.mgh.harvard.edu). The specific methods used for cortical reconstruction of T1‐MRI brain images have been fully described elsewhere. In short, optimized surface deformation models following intensity gradients accurately identify white matter and gray matter boundaries in the cerebral cortex, from which cortical thickness is computed at each vertex of the resulting surfaces. The resulting vertex‐wise Cth data are normalized to average space and smoothed using a Gaussian kernel of 15 mm FWHM.

### Statistical analysis

Data are presented as means and standard deviations for continuous variables and percentages for categorical variables. Statistical comparisons utilized independent t‐tests, MANOVA, and Mann–Whitney for continuous, ordinal, and categorical variables, respectively. Bivariate correlation linear regression or logistic regression analysis was employed, with age, gender, education, and CAG repeat length considered as nuisance variables in most analyses.

According to standard approaches, the cognitive status of the sample was categorized as cognitively preserved vs cognitively impaired based on the presence of two impaired tests in one domain (*z* < −1.5) or one test impaired on two domains.^40^ Then, we compared biomarker levels between these two groups. We also identified patients with a predominant impairment in fronto‐striatal‐dependent processes versus patients with additional posterior‐cortical deficits, and compared biomarker levels based on this classification.

Voxel‐wise and vertex‐wise measures derived from VBM and Cth analyses were introduced into a general lineal model (GLM) to explore the structural brain correlates of the different levels of CSF biomarkers. Again, these GLM included age and CAG length as nuisance covariates. Only clusters surviving *p* < 0.05 and family‐wise error (FWE) correction for multiple comparisons were considered significant. FWE correction was performed using cluster‐level RFT for the VBM analyses as implemented in SPM12, and a Monte Carlo simulation with 10,000 repeats was used for the Cth analysis as implemented in FreeSurfer. To investigate the potential associations between the biomarker‐related imaging measures and the performance in the different cognitive tasks, we computed average GMV and Cth values at the identified clusters where we observed significant effects. In a linear regression using the same covariates, we studied the association of these imaging measures with the scores obtained in each cognitive measure. Nonimaging analyses were performed using the statistical package SPSS v.23 (IBM SPSS Statistics for Windows, Version 23.0. Armonk, NY: IBM Corp), and a two‐sided p‐value<0.05 was considered significant. The data that support the findings of this study are available from the corresponding author, upon reasonable request. The data are not publicly available due to restrictions derived from the points established in the informed consent of the participants and in the approval by the ethics committee in relation to the transfer of data.

## Results

### Clinical and sociodemographic characteristics

The main sample of symptomatic participants consisted of 56% female and 43% male with a mean age of 48.8 (11.8) years, a mean CAG repeat length of 43.9 (2.7), with a mean TFC of 11.1 (2.5), and a mean CAP score of 486.5 (117). The sample of premanifest gene‐mutation carriers consisted of 54% female and 46% male, with a mean age of 37.7 (8.6) years, a mean CAG repeat length of 42.8 (2.1), and a mean CAP score of 342.45 (75.2). No differences were found between these groups in terms of CAG repeat length [*t*(42) = 1.5; *p* = 0.120]. Table 1 summarizes the main clinical and sociodemographic characteristics of the sample.

**Table 1:** Clinical, sociodemographic data and biomarker profile of the sample.

	preHD	Manifest HD	*p*
Age	37.7 (8.6)	48.8 (11.8)	<0.0001
Gender (f/m)	12/10	11/13	
Education (years)	13.5 (3.3)	11.7 (4.2)	0.117
CAG	42.8 (2.1)	43.9 (2.7)	0.105
CAP score	342.4 (75.2)	486.5 (117)	<0.0001
UHDRS‐TMS	0.4 (1.1)	25.6 (23.1)	<0.0001
cUHDRS	17.1 (2.7)	11.2 (5.1)	<0.0001
TFC	13	11.1 (2.5)	0.001
FIS	100	90.3 (13.1)	<0.0001
CSF NfL (pg/mL)	1016.1 (606.6)	2655.8 (1224.44)	<0.0001
CSF total Tau (pg/mL)	83.8 (36.5)	110.5 (43.4)	0.027
CSF pTau‐231 (pg/mL)	27.8 (10.2)	37.7 (15.3)	0.013

CAG, CAG repeat length; CAP score, CAG × AGE product; cUHDRS, Composite UHDRS; FIS, Functional Independence Score; TFC, Total Functional Capacity; UHDRS‐TMS, Unified Huntington's Disease Rating Scale Total Motor Score.

**Figure 1:**
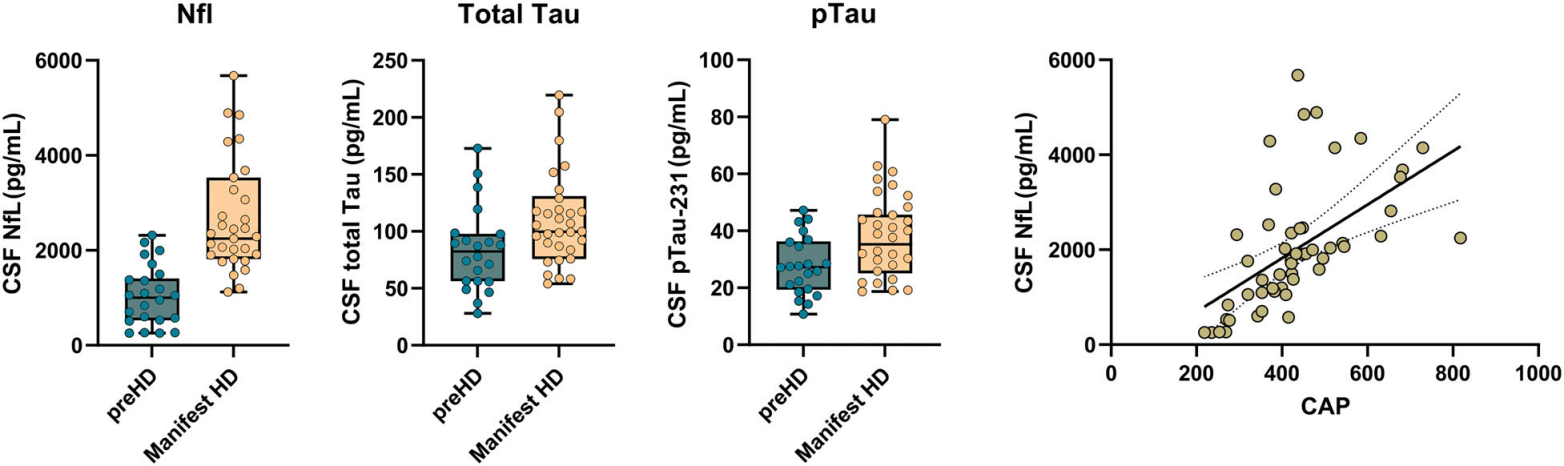
Biomarker profile of the initial sample. The figure shows the differences (*p* < 0.05) between premanifest and manifest HD groups in the biomarker's levels and global association with the CAP score.

Regarding biomarkers, CSF biomarkers were highly correlated between each other, maintaining the significance when controlling for age, CAG, and CAP score. Significant differences were found between groups in the form of greatly increased values in the group of symptomatic participants for NfL [*t*(42) = −6.6; *p* < 0.0001], total Tau [*t*(42) = −2.5; *p* = 0.015], and pTau‐231 [*t*(42) = −2.6; *p* = 0.012] (Fig. 1). Correlations in the premanifest group showed a highly significant relationship between NfL levels and CAP score (*r* = 0.751; *p* < 0.0001) and with age (*r* = 0.594; *p* = 0.004).

The classification of manifest participants according to cognitive status revealed 15 (50%) with relatively preserved cognition (NC‐HD) and 15 (50%) with impaired cognition (IC‐HD). We use the term “relatively” because in all cases, there was involvement of at least one test related to executive frontal function, processing speed, or attention.

Congruent with this classification, there was a significant difference in the total PD‐CRS between groups [*t*(30) = 4.26; *p* < 0.0001], with a mean PD‐CRS total score of 95 (17) in the NC‐HD group and of 65.3 (15.9) in the IC‐HD group. No differences were found in relation to CAP score, age, or educational level. As expected, the NC‐HD group scored significantly better in TFC [*t*(30) = 2.34; *p* = 0.036]. Among the sample, 17.4% showed impairment in one cognitive domain and the other 82.6% showed impairment in two or more cognitive domains. Processing speed was the most prevalently compromised process (55.2%), followed by working memory (44%), executive functions (36%), memory (33.3%), language (29.2%), and visuoperceptual/spatial and constructive processes (24%). Of the total sample, 38% presented altered performance on one frontal‐executive, attention or processing speed task or fulfilled criteria for a single‐domain (frontal‐executive) cognitive impairment. The other 62% presented a multi‐domain form of cognitive impairment with compromise of memory, language, and visuoperceptual/spatial processes superimposed to frontal‐related deficits.

### Associations between biomarkers and cognition

**Figure 2:**
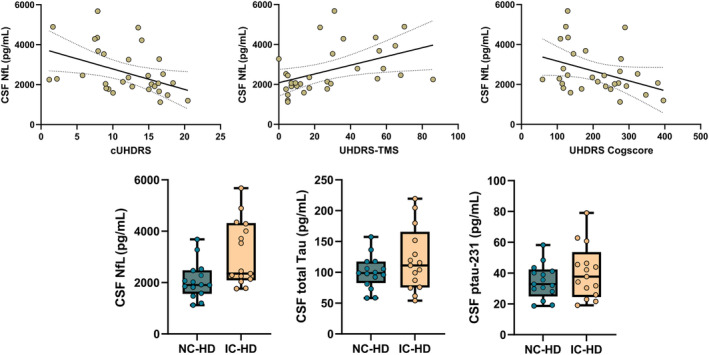
Biomarker differences in subjects with and without cognitive impairment. The figure shows the differences (*p* < 0.05) in biomarker's levels between different cognitive status and the associations between biomarkers and clinical parameters. NfL: NC‐HD = 1614.96 pg/mL vs IC‐HD = 3116.08 pg/mL; tTau: NC‐HD = 92.31 pg/mL vs IC‐HD = 121.35 pg/mL; pTau: NC‐HD = 31.39 pg/mL vs IC‐HD = 41.56 pg/mL. NC‐HD refers to “normal cognition” and IC‐HD refers to “impaired cognition”.

In the sample of manifest participants, CSF biomarkers were highly correlated between each other, maintaining the significance when controlling for age, CAG, and CAP score. No associations were found between CAG, CAP score, and CSF levels of tTau and pTau‐231, but this latter showed a significant relationship with age (*r* = 0.532; *p* = 004). NfL levels were significantly associated with lower cUHDRS (*r* = −0.442; *p* = 0.018), UHDRS cogscore (*r* = −0.422; *p* = 0.025), and higher UHDRS‐TMS (*r* = 0.453; *p* = 0.015). Levels of total tau and pTau‐231 showed a similar pattern of association with cUHDRS and UHDRS‐TMS, but significance did not survive after controlling for age (Fig. 2, Table S2).

The MANOVA analysis performed between IC‐HD and NC‐HD groups using CAG, age, and gender as covariables revealed significant differences in favor of higher NfL levels [*F*(3, 21) = 6.7, *p* = 0.016, *η* ^2^ = 0.235], pTau‐231 levels [*F*(3, 21) = 5.1, *p* = 0.034, *η* ^2^ = 0.188], and total Tau levels [*F*(3, 21) = 5.63, *p* = 0.027, *η* ^2^ = 0.204] in the IC‐HD. In order to further explore the contribution of the different biomarkers to the cognitive phenotype, we performed a t‐test comparison between the single‐domain vs. multi‐domain groups. No differences were found for age, CAG repeat length, or CAP score between these groups. Significant differences were found regarding CSF biomarkers indicating increased NfL [*t*(30) = 3.6; *p* = 0.009], total Tau [*t*(30) = 2.3; *p* = 0.02], and pTau‐231 [*t*(30) = 2.5; *p* = 0.01] in the multi‐domain group.

Linear regression analysis focusing on the association between biomarker levels and the specific tasks comprising each cognitive domain showed a significant negative association between NfL levels and performance in several tasks with clear dependence on processing speed, executive functions, and working memory, such the Stroop color (*β* = −0.511; *p* = 0.013) and word‐naming test (*β* = −0.502; *p* = 0.015), the SDMT (*β* = −0.543; *p* = 0.007), and the part A of the TMT (*β* = −0.463; *p* = 0.026).

**Figure 3:**
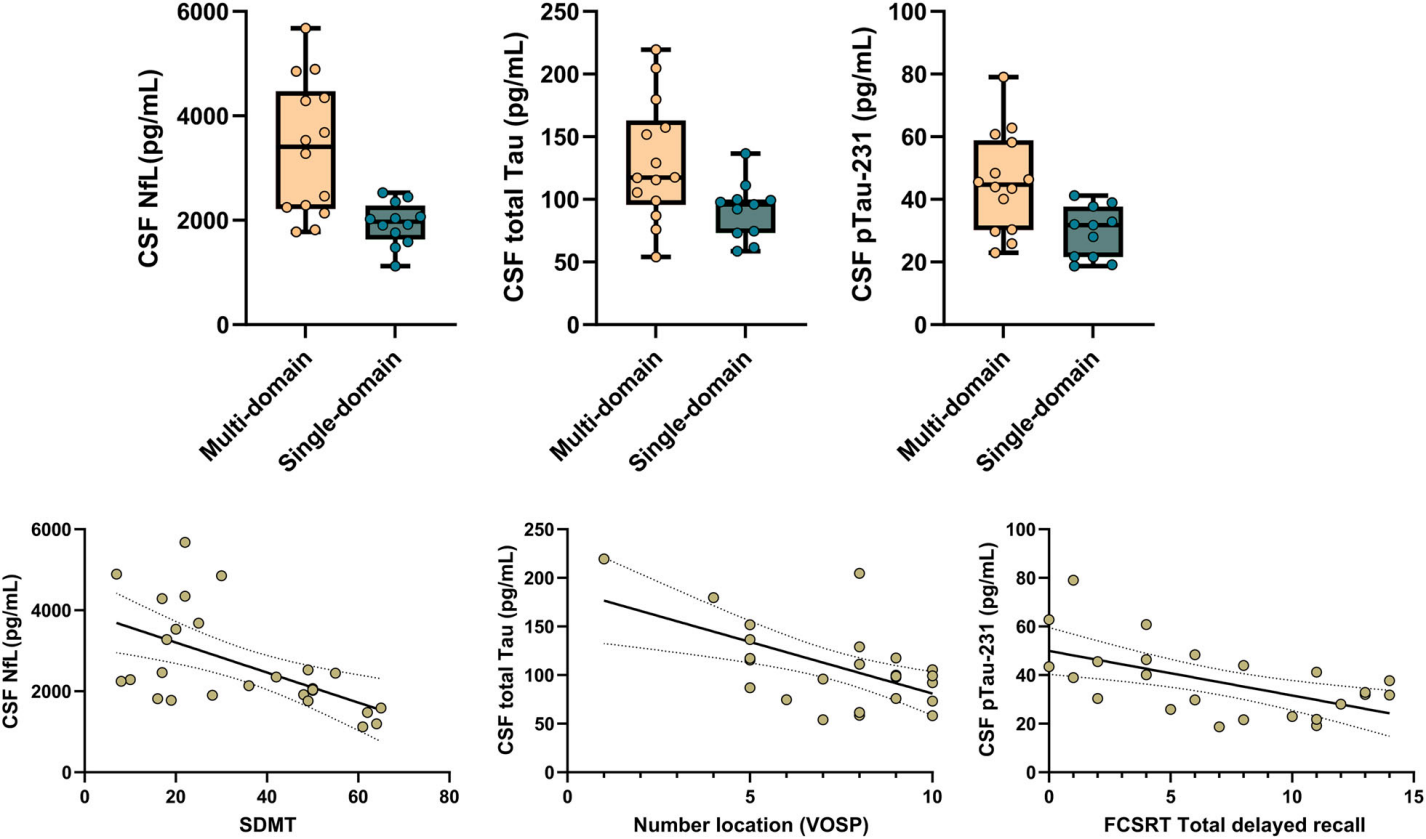
Biomarker differences as a function of cognitive impairment profile. The figure shows differences (*p* < 0.05) in biomarker's levels as a function of the cognitive profile of cognitive impairment in terms of single vs multi‐domain. Associations with specific cognitive measures are also shown.

Significant negative associations were found between total Tau levels and performance in the semantic verbal fluency test (*β* = −0.466; *p* = 0.035), the Benton Facial Recognition (*β* = −0.472; *p* = 0.031), and the position discrimination (*β* = −0.475; *p* = 0.040) and Number location (*β* = −0.591; *p* = 0.008) VOSP subtests. For pTau‐231, significant negative associations were found with performance in the forward Digit Span (*β* = −0.493; *p* = 0.032), the VOSP Number location subtest (*β* = −0.524; *p* = 0.021), and the total delayed recall from the FCSRT (*β* = −0.516; *p* = 0.012) (Fig. 3).

### Association between biomarkers and neuroimaging parameters

The voxel‐wise regression analysis revealed an association between higher NfL levels and lower GMV in several cortical and subcortical regions, including the caudate nucleus, the angular, mid temporal, mid frontal, medial frontal, and the lingual gyrus (table reporting GMV clusters can be found in Supplementary Data). NfL‐related GMV measures showed, in turn, significant associations with a set of cognitive measures previously found to be associated with NfL. Among them, stands out the association between GMV at the caudate nucleus and SDMT (*r* = 0.688; *p* < 0.0001), SWRT (*r* = 0.551; *p* = 0.008), UHDRS cogscore (*r* = 0.594; *p* = 0.004), and cUHDRS (*r* = 0.702; *p* < 0.0001). Significant relationships were also found between the right superior temporal and SDMT (*r* = 0.661; *p* = 0.001), cUHDRS (*r* = 0.647; *p* = 0.001), and UHDRS cogscore (*r* = 0.572; *p* = 0.005), as well as between the lingual gyrus and all the tasks with a visual component.

**Figure 4:**
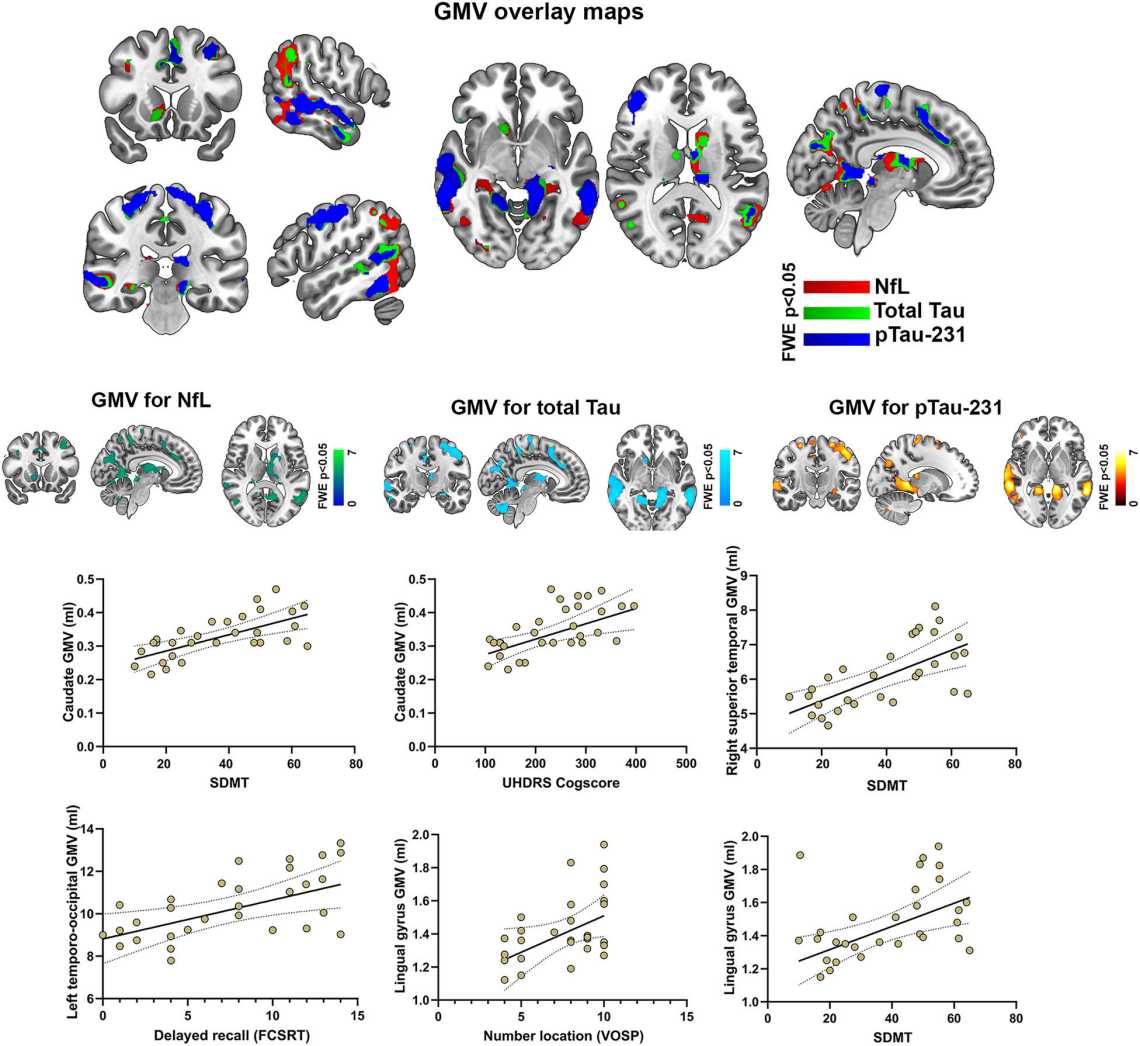
Correlates in GMV of different biomarker levels and clinical parameters. (top) VBM‐GMV correlates of NfL, tTau and pTau (*p* < 0.05 corrected), including an overlay map. (bottom) Scatter plots illustrating the associations between the biomarker‐related imaging alterations and cognitive performance.

Regarding Tau and pTau‐231, associations were found between temporo‐occipital regions and the SDMT (*r* = 0.651; *p* = 0.001), all Stroop test components (*r* = 0.563; *p* = 0.006), cUHDRS (*r* = 0.615; *p* = 0.002), and UHDRS cogscore (*r* = 0.555; *p* = 0.007). For these biomarkers, other correlations that were not found in relation to NfL were identified. Among them, some of the most representative were found at the level of GMV in the temporal gyrus and episodic memory performance in terms of free (*r* = 0.575; *p* = 0.006) and cued recall (*r* = 0.594; *p* = 0.005), visuoperceptive performance in JLOT (*r* = 0.591; *p* = 0.005), and TMT‐B (*r* = −0.638; *p* = 0.001). Moreover, GMV at the lingual gyrus was significantly associated with processes with a visual component. The most notable ones were the SDMT (*r* = 0.575; *p* = 0.005), the JLOT (*r* = 0.625; *p* = 0.002), and the position discrimination subtest of the VOSP (*r* = 0.484; *p* = 0.02) (Fig. 4).

**Figure 5:**
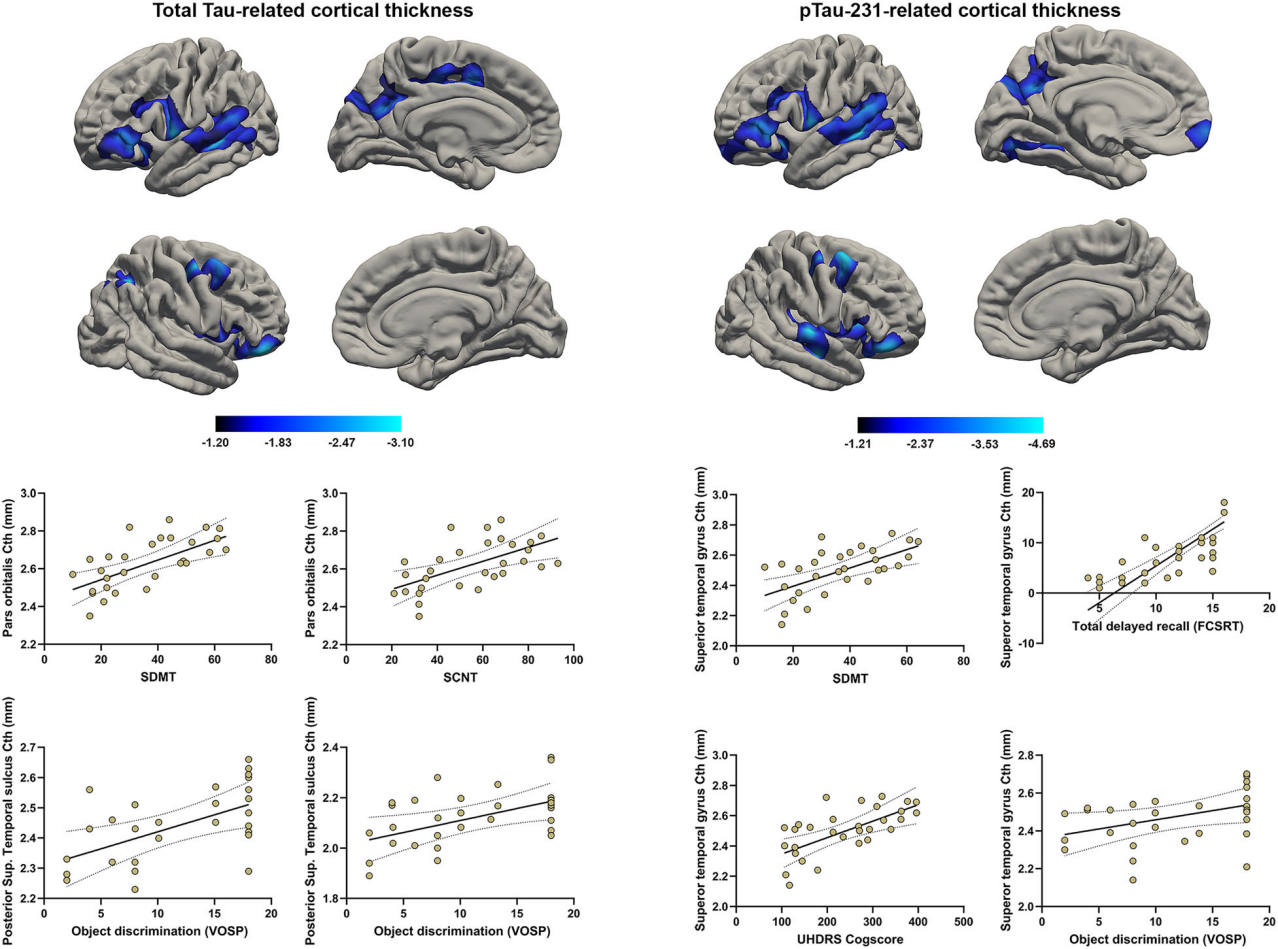
Correlates in Cth of different biomarker levels and clinical parameters. (top) Vertex‐wise Cth correlates tTau and pTau (*p* < 0.05 corrected). No significant clusters were found in the NfL GLM analysis. (bottom) Scatter plots illustrating the associations between the biomarker‐related cortical thinning and cognitive performance.

In the vertex‐wise regression analysis of Cth data, there was no significant association between NfL levels and cortical thinning. In contrast, total Tau levels were associated with decreased Cth in extensive left fronto‐temporo‐parieto‐occipital territories and right fronto‐parietal regions, with notable involvement of the posterior region of the superior and middle temporal gyrus, inferior orbitofrontal cortex, temporo‐parietal junction, dorsolateral prefrontal cortex, precuneus, cuneus, insular cortex, and middle frontal cortex. The maps obtained in relation to pTau‐231 levels largely overlapped with those obtained for total Tau, but they highlighted a greater involvement of the anterior region of the right superior temporal cortex and a greater extension of the left temporo‐parietal and occipital thickness (Fig. 5).

Total Tau‐related thinning at the left posterior region of the superior temporal sulcus was associated with lower performance on the UHDRS cogscore (*r* = 0.523; *p* = 0.045), VOSP position discrimination subtest (*r* = 0.610; *p* = 0.016), and Benton's Facial Recognition Test (*r* = 0.533; *p* = 0.041). Cth values at the pars orbitalis were associated with lower performance in a set of frontal‐dependents tasks such as the SWRT (*r* = 0.615; *p* = 0.018), the SDMT (*r* = 0.648; *p* = 0.009), and the UHDRS cogscore (*r* = 0.688; *p* = 0.005). Cortical integrity at both the precuneus and superior parietal regions was associated with lower performance on the object discrimination subtests of the VOSP (*r* = 0.600; *p* = 0.018). Regarding pTau‐related thickness, results from the more posterior region on the superior temporal sulcus mostly overlapped with those found for total Tau, showing an association with lower performance on the object discrimination subtest of the VOSP (*r* = 0.651; *p* = 0.009). The cluster found at level of the insular cortex was associated with lower performance on the SWRT (*r* = 0.527; *p* = 0.044) and on the UHDRS cogscore (*r* = 0.584; *p* = 0.022), and the cluster at level of the superior temporal cortex was strongly associated with lower performance on the SWRT (*r* = 0.604; *p* = 0.017), SDMT (*r* = 0.659; *p* = 0.008), and UHDRS cogscore (*r* = 0.653; *p* = 0.008). Moreover, the pTau‐related cluster in the superior temporal correlated with total delayed recall in the FCSRT (*r* = 0.638; *p* = 0.014) and with phonemic fluency (*r* = 0. 606; *p* = 0.020).

## Discussion

In the present study, we explored the contribution of NfL, total Tau and pTau‐231 on the expression of cognitive deficits and on the trajectory of neurodegenerative brain changes in early‐to‐middle manifest HD.

Our results highlight that, although processing speed and frontal‐executive‐related deficits are prominently characteristic of HD, visuoperceptive and language‐related deficits are significantly impaired in a subset of individuals, without age, CAG repeat length, or disease duration being the mechanisms that explain the presence of these deficits. These alterations, in addition to the eminently frontal‐executive ones, were related to a more aggressive cognitive phenotype, emphasizing the previously reported relationship between cortical‐posterior alterations and more severe forms of cognitive progression in HD.^2^, ^31^

At a biomarker level, NfL were related to processing speed and other frontal‐related measures that have been shown in multiple studies to be highly sensitive to change throughout HD progression.^6^, ^41^, ^42^ Interestingly, NfL levels showed no relationship with a set of cognitive alterations that appeared characteristic of the subgroup of cases with greater cognitive compromise. This suggests that NfL levels are possibly not sensitive to a subtype of deficits that may not occur in all patients equally and that define more aggressive cognitive phenotypes.

Regarding total Tau and pTau‐231 levels, we found that, unlike what was observed for NfL levels, these biomarkers do show a clear relationship with the alterations described in the visuoperceptive and language domains in the more severe cognitive phenotype. The fact that not all patients present alterations in these domains, and the lack of relationship observed between NfL levels leads us to think that total Tau and pTau‐231 possibly contribute to the cognitive phenotype of HD in a subgroup of cases, but not in all of them.

The lack of correlation between CAG repeat length, the disease burden, and the levels of total Tau and pTau‐231 suggests that the triggering mechanism of Tau‐related pathology in HD does not necessarily depend on the size of the expansion or the cumulative livelong exposure to the mutation. Hence, it becomes intriguing to explore the potential of investigating the sequence of biological events that may contribute to the expression of this type of Tau‐related pathology in HD.^43^ In any case, given the age and characteristics of the subjects, it does not seem reasonable to assume that these Tau levels are secondary to the fact that they also have tauopathy or Alzheimer's disease.

Overall, the results indicate that neurocognitive impairment in HD may manifest in various cognitive forms, much like it is observed in other neurodegenerative conditions with which distinct cognitive phenotypes. These different cognitive forms in HD are likely influenced by the combined effects of various factors, including added Tau‐related pathology, in addition to the mechanisms already inherent to HD and those that have subsequently proven to be critical in expressing their heterogeneity.^20^, ^44^, ^45^, ^46^

Regarding neuroimaging analysis, our cohort revealed a distinct pattern of biomarker associations. Specifically, NfL levels did not show a significant association with cortical thickness.^47^ Instead, they were found to be related to GMV in several brain regions, including the caudate and putamen, medial and dorsolateral frontal regions, superior and middle temporal gyrus regions, and to some extent, certain occipital regions. Conversely, Tau and pTau‐231 levels exhibited a clear relationship with Cth and also with GMV in extensive fronto‐temporo‐parietal, occipital, and subcortical regions. Consistent with these findings, measurements of Cth and/or GMV more related to levels of the examined biomarkers were closely linked to cognitive test performance. Particularly, tests that relied more on NfL levels showed a strong association with the structural integrity of the specific brain regions that are linked to NfL levels in this study. Similarly, tasks that relied more on Tau levels also showed a clear association with the structural integrity of brain regions associated with Tau. In our opinion, these findings provide consistent biological and conceptual plausibility to a model that links the effects of different biomarkers on structural integrity and related cognitive performance.

Identifying cognitive phenotypes in neurodegenerative diseases is a challenge that requires exploring in‐depth cognitive functioning. This approach has revealed different cognitive phenotypes within the same diseases,^46^ as is the case for posterior‐cortical atrophy and logopenic variants of AD, or for primary progressive aphasias and the behavioral variant of frontotemporal degenerations, as well as for the whole spectrum of atypical parkinsonisms.^45^ Although efforts to explore different cognitive phenotypes in HD have been limited, a previous study from our group already demonstrated two major cognitive phenotypes during the early years of progression associated with a more prominent pattern of temporo‐parieto‐occipital atrophy.^2^ More recently, our group demonstrated that during the first 3 years of HD progression, two major cognitive phenotypes, in terms of fast and slow progressors, can be identified, regardless of disease burden.^1^ Unfortunately, the available data in this previous study did not allow for a deeper investigation into the biological mechanisms that could be associated with each group.

The current data incorporate additional knowledge regarding the mechanisms associated with these phenotypes, highlighting the role of Tau‐related pathology. However, there are other mechanisms whose modifying role in disease expression is widely recognized. Among them, somatic instability in different types of cells has gained special relevance because of its role on the neuropathological and clinical variability of HD.^20^ In this sense, our results are not intended to replace the role of these other mechanisms, but reinforce the idea that, similar to other neurodegenerative processes, although a single biological mechanism triggers HD, there are several additional contributors to the clinical spectrum of the disease.

Undoubtedly, it is worth considering the mechanisms through which HD could promote Tau‐related pathology. The study of these mechanisms has been the subject of important research in the field of HD, which allows us to delve into the processes through which the genetic mutation, the mHTT protein itself, and obviously, neurodegeneration, could contribute to modulating tau levels and tau hyperphosphorylation.^26^, ^44^ Gaining a deeper understanding of these mechanisms from the field of molecular and systems biology is an essential task to be pursued in the near future.

The present study is not without limitations. Firstly, it is unquestionable that the work has been carried out with a small sample and that these results deserve to be replicated and extended to a larger cohort. Moreover, investigating the presence of Tau pathology in the brain using postmortem tissue and positron emission tomography tracers specific for Tau would allow for a much more robust understanding of the presence and distribution of Tau in the HD brain.^48^, ^49^ Additionally, we are aware of the limitations that may arise from using a specific pTau epitope since it is possible that the form pTau takes in HD may be more or less sensitive to quantifications using some specific epitopes.^37^, ^38^ Of course, we are also aware that exploring the role of these biomarkers in premanifest population, where we know the disease trajectory can be highly variable, would be worthwhile. In any case, we believe that the study we have conducted, despite its limitations, demonstrates the role of Tau‐related pathology in HD and its involvement in modulating the cognitive phenotype.

In conclusion, in‐depth neurocognitive assessment of HD allows for the identification of phenotypes that combines alterations in visuoperceptual and language functions onto a predominantly represented frontal‐executive and striatal‐dependent deficits. These alterations are consistent with a more severe neurocognitive syndrome. The presence of these alterations is accompanied by significantly higher CSF levels of total Tau and pTau‐231, which in turn are related to a more pronounced pattern of posterior‐cortical atrophy. This pattern implicates a set of brain territories closely linked to the cognitive processes affected by Tau. All of this reinforces the notion that there is an association between Tau‐related pathology, cognitive impairment, and neurodegeneration in HD.

## Author Contributions

SMH: (1) conception and design of the study, (2) acquisition and analysis of data, or (3) drafting a significant portion of the manuscript or figures. JPP: (1) conception and design of the study, (2) acquisition and analysis of data, or (3) drafting a significant portion of the manuscript or figures. RPG: (2) acquisition and analysis of data or (3) drafting a significant portion of the manuscript. FS: (2) acquisition and analysis of data or (3) drafting a significant portion of the manuscript. AHB: (2) acquisition and analysis of data or (3) drafting a significant portion of the manuscript. AC: (2) acquisition and analysis of data or (3) drafting a significant portion of the manuscript. ERA: (2) acquisition and analysis of data or (3) drafting a significant portion of the manuscript. APD: (2) acquisition and analysis of data or (3) drafting a significant portion of the manuscript. JP: (1) conception and design of the study, (2) acquisition and analysis of data, or (3) drafting a significant portion of the manuscript or figures. JK: (1) conception and design of the study, (2) acquisition and analysis of data, or (3) drafting a significant portion of the manuscript or figures.

## Conflict of Interest

SMH has received honoraria for lecturing from Teva, Zambon, UCB, and Roche, and reports grants from Huntington's disease Society of America (Human Biology Project), and from Fondo de Investigaciones Sanitarias (FIS) from Instituto de Salud Carlos III (ISCIII). JPP reports a grant from Fondo de Investigaciones Sanitarias (FIS) from Instituto de Salud Carlos III (ISCIII). JK has received honoraria for advisory boards or lecturing from: Teva, Zambon, UCB, Bial, General Electric, Sanofi, and Roche, and reports grants from Fundació la Marato de TV3, Fondo de Investigaciones Sanitarias (FIS) from Instituto de Salud Carlos III (ISCIII), and Fondo Europeo de Desarrollo Regional (FEDER). JP has served on advisory or speakers' boards, and received honoraria from UCB, Zambon, AbbVie, Italfarmaco, Allergan, Ipsen, and Bial and reports grants from Fundació la Marato de TV3, Fondo de Investigaciones Sanitarias (FIS) and from Instituto de Salud Carlos III (ISCIII). The authors declare that there is no conflict of interest regarding the publication of this article.

## Supporting information


Table S1.

## Data Availability

Researchers at a recognized research organizations can open an account and request data from the used Enroll‐HD dataset via the process outlined in the Enroll‐HD website regarding data sharing and biosamples: https://enroll‐hd.org/for‐researchers/access‐data/.
