# Supplementary material for: Cognitive phenotype and neurodegeneration associated with Tau in Huntington's disease
Source: Ann Clin Transl Neurol. 2024 Mar 27;11(5):1160–71. doi: 10.1002/acn3.52031 (PMC11093246; doi:10.1002/acn3.52031)
Supplement: Supplementary file 1 — Table S1. [file ACN3-11-1160-s001.pdf]

Supplementary data:

Supplementary table 1: Cluster description table of the VBM-GMV analyses

NfL levels	MNI coordinates (x, y, z)	Cluster size (voxels)	*T* value
Left angular gyrus	-45 -54 35	10210	7.91
Left mid temporal	-62 -51 0		7.05
Left lingual	-9 -35 -9		6.15
Left postcentral	-23 -18 52	1635	6.86
Right mid temporal	66 -48 -6	4128	6.42
Right superior temporal	69 -18 -3		5.74
Left mid frontal	-39 14 56	1461	6.06
Left medial frontal superior	0 32 43	832	4.56
Precuneus	-2 -42 57	924	5.97
Right lingual	23 -33 11	1972	5.67
Right angular	53 -54 32	1547	5.45
Caudate nucleus	-9 6 18	1347	4.49
Total Tau / pTau leves	MNI coordinates (x, y, z)	Cluster size	*T* value
Left lingual	-15 -44 2	6813	6.66
Left mid temporal	-62 -50 3		6.39
Left fusiform gyrus	-43 -48 -20		6.25
Left parahippocampal gyrus	-17 -36 -9		6.14
Right mid temporal	59 -42 -2	2902	6.53
Supplementary motor area	11 -21 68	8051	6.52
Left precentral gyrus	-23 -18 62		6.44
Left mid frontal gyrus	-42 21 45		5.79
Right lingual	11 -53 -6	942	5.74
Right precentral gyrus	24 -27 65	1165	5.36
Left occipital superior	-20 -71 33	1183	5.19
Right angular gyrus	50 -53 33	807	4.64
Left hippocampus	-26 -12 -14	707	4.24
Right hippocampus	29 -20 -14	703	4.08
Right superior temporal	63 -20 1	8031	6.54

Supplementary table 2: Association between CSF biomarkers, sociodemographic, clinical and cognitive variables

Variables	NfL	tTau	pTau-231
NfL	1	-	-
tTau	0.808	1	-
pTau-231	0.836	0.887	1
Age	ns	ns	0.532
CAG	ns	ns	ns
CAP score	ns	ns	ns
UHDRS-TMS	0.453	ns	ns
UHDRS cogscore	-0.422	ns	ns
cUHDRS	-0.442	ns	ns
SCNT	-0.511	ns	ns
SWRT	-0.502	ns	ns
SDMT	-0.543	ns	ns
TMT-A	-0.463	ns	ns
Semantic verbal fluency	ns	-0.466	ns
BFRT	ns	-0.472	ns
PD-VOSP	ns	-0.475	ns
NL-VOSP	ns	-0.591	-0.524
Forward Digit span	ns	ns	-0.493
FCSRT Total DR	ns	ns	-0.516

BFRT: Benton Facial Recognition Test. SCNT: Stroop color-naming test, SWRT: Stroop word-reading test. SDMT: Symbol Digit Modalities Test. TMT-A: Trail making test part A. PD-VOSP: Position discrimination subtests of the Visual and Object Shape Perception test. NL-VOSP: Number location subtests of the Visual and Object Shape Perception test. FCSRT Total DR: Free and Cued Selective Reminding Test total delayed recall.
